# Freezing first: insights from 8 years of planned oocyte cryopreservation at an “egg freezing clinic”

**DOI:** 10.1016/j.fertnstert.2025.12.003

**Published:** 2025-12-05

**Authors:** Joshua U. Klein, Nicholas B. Conway, Baruch Abittan, Shelby Marcinyshyn, Valerie Shafran, Dayna Hennessy, Jonathan Lo, Dawn Kelk, Anat Chemerinski, Patricia Greenberg, Nataki C. Douglas

**Affiliations:** a Extend Fertility, New York, New York; b Division of Reproductive Endocrinology and Infertility, Department of Obstetrics, Gynecology and Reproductive Health, Rutgers Health, New Jersey Medical School, Newark, New Jersey; c Rutgers School of Public Health, Piscataway, New Jersey

**Keywords:** Oocyte cryopreservation, egg freezing, metaphase II oocyte, MII oocyte, oocyte warming, fertility preservation

## Abstract

**Objective:**

To assess the outcomes of oocyte warming (OW) and embryo transfer in individuals who underwent planned oocyte cryopreservation (OC).

**Design:**

Retrospective cohort study.

**Subjects:**

This study includes all planned OC cycles performed at Extend Fertility before December 31, 2023, and subsequent OW cycles performed before August 2024.

**Exposure:**

Age at the time of OC and number of metaphase (M)II oocytes warmed.

**Main Outcome Measures:**

The primary outcome was cumulative ongoing pregnancy/live birth (OP/LB) rate per OW cycle, stratified by age and number of MII oocytes warmed. Secondary outcomes included OW survival and fertilization rates and the number of euploid embryos per MII oocyte, categorized by age at the time of OC.

**Results:**

Between 2016 and 2023, 4,659 OC cycles were completed for 3,138 patients, with the mean OC start age decreasing from 36.9 ± 2.8 years at the onset to 35.0 ± 3.5 years in the final year of the study. The mean length of time between OC and first OW was 3.4 ± 1.9 years. The mean age at first OW was 39.9 ± 3.5 years, with a median (interquartile range) of 40 (39, 42) years. Among patients who had completed at least one OC cycle before 2020 (n = 2,163), allowing for a minimum follow-up period of 4 years, 10.4% (n = 226) returned for OW. Across 271 OW cycles, the mean number of MII oocytes warmed was 15, with survival and fertilization rates of 90.7% and 77.2%, respectively. Among patients who underwent OC at ≤40 years of age, warming a higher number of MII oocytes was associated with increased euploid embryo yield. The cumulative ongoing pregnancy/live birth rate (OP/LB) rate for the cohort was 70.3%, ranging from 58.3% for 1–9 MII oocytes warmed to 81.8% for >20 MII oocytes warmed.

**Conclusion:**

Driven by increased awareness and access, patients are undergoing planned OC at younger ages. This study demonstrates high success rates achieved at an “egg freezing clinic”, especially among patients who pursue OC at younger ages. It also establishes a framework to estimate the number of euploid embryos from MII oocytes, as well as the likelihood of live birth, stratified by age at OC.

## Abstract (es)

**Objetivo:**

Evaluar los resultados de la descongelación de ovocitos (DO) y de la transferencia embrionaria (TE) en personas que se sometieron a criopreservación planificada de ovocitos (CPO).

**Diseño:**

Estudio de cohorte retrospectivo.

**Sujetos:**

Este estudio incluye todos los ciclos de CPO realizados en Extend Fertility antes del 31 de diciembre de 2023, y los ciclos posteriores de DO realizados antes de agosto de 2024.

**Exposición:**

Edad en el momento de la CPO y número de ovocitos en metafase II (MII) descongelados.

**Medidas principales de resultado:**

El resultado primario fue la tasa acumulada de embarazo evolutivo/nacido vivo (EE/NV) por ciclo de DO, estratificada por edad y por número de ovocitos MII descongelados. Los resultados secundarios incluyeron las tasas de supervivencia y de fecundación tras la DO, y el número de embriones euploides por ovocito MII, categorizados por la edad en el momento de la CPO.

**Resultados:**

Entre 2016 y 2023 se completaron 4.659 ciclos de CPO en 3.138 pacientes, disminuyendo la edad media al inicio de la CPO de 36,9 ± 2,8 años al comienzo a 35,0 ± 3,5 años en el último año del estudio. El tiempo medio entre la CPO y la primera DO fue de 3,4 ± 1,9 años. La edad media en la primera DO fue de 39,9 ± 3,5 años, con una mediana (rango intercuartílico) de 40 (39, 42) años. Entre las pacientes que habían completado al menos un ciclo de CPO antes de 2020 (n = 2.163), lo que permitió un seguimiento mínimo de 4 años, el 10,4% (n = 226) regresó para DO. En 271 ciclos de DO, el número medio de ovocitos MII descongelados fue de 15, con tasas de supervivencia y fecundación del 90,7% y 77,2%, respectivamente. En pacientes que realizaron la CPO a ≤ 40 años, la descongelación de un mayor número de ovocitos MII se asoció con un mayor rendimiento de embriones euploides. La tasa acumulada de EE/NV para la cohorte fue del 70,3%, oscilando entre 58,3% para 1–9 ovocitos MII descongelados y 81,8% para >20 ovocitos MII descongelados.

**Conclusión:**

Impulsadas por una mayor concienciación y acceso, las pacientes están realizando la CPO a edades más tempranas. Este estudio demuestra altas tasas de éxito logradas en una “clínica de congelación de óvulos”, especialmente entre pacientes que optan por la CPO a edades más jóvenes. Además, establece un marco para estimar el número de embriones euploides a partir de ovocitos MII, así como la probabilidad de nacido vivo, estratificando por la edad en el momento de la CPO.

In the past half-century, there has been a major shift in the average age at first childbirth (1). For example, among women in the United States, the average age at first birth increased from 21.4 years in 1970 to 27.3 years in 2023 (2, 3). Because of the well-characterized age-related decline in fertility, women who delay childbearing may encounter increased difficulty conceiving at older ages. Although assisted reproductive technologies (ART) increase the odds of conception at advanced ages, the success rates of in vitro fertilization (IVF) are universally relatively low for women in their late 30s, and extremely low for women in their early-mid 40s (4). Over the past decade, planned oocyte cryopreservation (OC) has become a widely accepted method for preserving reproductive potential in younger women delaying childbearing (5–7).

Fertility preservation has evolved significantly over the years. In 2006, the American Society of Clinical Oncology first recommended fertility preservation techniques for women diagnosed with malignancies or undergoing gonadotoxic therapy (8). By 2012, the American Society for Reproductive Medicine (ASRM) reclassified OC as a nonexperimental procedure (8, 9). Subsequently, in 2014, ASRM recognized fertility preservation as a strategy for women facing age-related fertility decline, and in 2018, the associated ethical considerations were addressed (5, 10). This was also supported by the European Society of Human Reproduction and Embryology in 2020 (11). The technology has seen rapid adoption; in the United States, OC cycles increased by 880% between 2010 and 2016 and by an additional 250% between 2017 and 2021 (2, 3). These OC cycles now account for almost 10% of ART cycles initiated in the United States (12).

However, despite the increased use of planned OC, the availability of data on the reproductive potential of cryopreserved autologous oocytes has not kept pace. A recent ASRM Ethics Committee Opinion (13) emphasized the importance of counseling patients regarding oocyte warming (OW) outcomes: “Providers should disclose their own clinic-specific statistics, or lack thereof, for successful freeze-thaw and live births.” Although many factors have likely contributed to the dearth of this data, perhaps the most significant barrier to the study of outcomes of cryopreserved oocytes is the relatively low rate of return, which limits the study of birth outcomes. In the United States, it is estimated that 10.8% of women have returned to use their cryopreserved oocytes. In a recent systematic review and meta-regression analysis, which included studies from the United States, Australia, and New Zealand, eight of ten studies reported return rates for oocyte utilization (14). A total of 1,517 patients returned to warm their cryopreserved oocytes, reflecting an average return rate of 11.1% ± 4.7%. Only four of ten studies reported ages at OC and OW, with an average age of 38.1 years at OC and an average age of 41.8 years at OW, demonstrating utilization of frozen oocytes within an average of 3.8 years after planned OC (14).

A review of the literature demonstrates that although there are at least 457 programs in the United States offering OC (15), fewer than five have published peer-reviewed data regarding OW outcomes (16–19). Given the limited data available on outcomes from warmed oocytes, patient counseling regarding the expected outcomes of OW is inherently constrained. When many programs offer a treatment without a proven record of successful outcomes, trust in our specialty could be undermined.

In this study, we review the 8-year experience with OC and OW at Extend Fertility. We believe it may be of particular interest as this center was initially established as an “egg freezing clinic” and subsequently evolved to provide comprehensive care for both infertility treatment and fertility preservation. We report trends in OC and outcomes after OW and embryo transfer. Our primary objective was to evaluate the outcomes of individuals who underwent autologous OW after planned OC, and to leverage these findings to improve patient counseling.

## MATERIALS & METHODS

### Design

With the approval of the BRANY Institutional Review Board (File #24–12-189–1680), the electronic medical record system eIVF (PracticeHwy.com Inc, Irving TX) at Extend Fertility from January 1, 2016, to August 1, 2024, was queried. This retrospective cohort study included patients who had planned OC cycles performed before December 31, 2023, and all OW cycles performed before August 2024. Demographic data, diagnosis and treatment protocol, and OC cycle outcomes were collected. Patients aged 18 to 46 years were included in the analysis. The maximum age for anti-müllerian hormone (AMH) testing was 45 years. Two individuals who underwent AMH testing at age 45 and subsequently completed a planned OC cycle at age 46 were included. Patients were excluded if OC was performed for a medical indication or because of a lack of sperm on the day of the oocyte retrieval.

### Ovarian stimulation, oocyte cryopreservation, warming, and embryo transfer

Ovarian stimulation protocol and starting doses were chosen by the treating physician and medication adjustments were made on the basis of ovarian response. Oocyte retrieval was performed 36 hours after oocyte maturation trigger administration. Immediately after the retrieval procedure, cumulus-oocyte complexes were denuded, and oocyte maturity was determined. Mature and late-maturing oocytes were vitrified approximately 2 hours after denudation. Kitazato vitrification media was used to perform oocyte vitrification as per manufacturer-specified protocols (20).

Oocyte warming was performed, as requested by the patient. All mature oocytes that survived the warming process were fertilized using standard intracytoplasmic injection (ICSI) protocols. After injection, oocytes were cultured and assessed approximately 18 hours after ICSI for the presence of two pronuclei. On day 2, cleavage assessment and laser-assisted hatching were performed on all embryos, after which developing embryos were transferred to new culture dishes. Embryos were graded on days 5, 6, and 7 using a modified Gardner system. Preimplantation genetic testing for aneuploidy (PGT-A) was discussed with all patients. For those who elected to proceed, trophectoderm biopsy was performed on blastocysts deemed suitable for testing. PGT-A was performed with next-generation sequencing.

For embryo transfer, the treating physician selected the endometrial preparation protocol on the basis of patient characteristics. For all patients, a saline infusion sonogram was conducted to ensure the uterine cavity was suitable for embryo transfer. In programmed cycles, patients received oral estradiol at increasing doses for at least 10 days to achieve endometrial thickness >7 mm, after which a combination of oral progesterone (200 mg twice daily), vaginal progesterone (200 mg twice daily), and progesterone in oil (50 mg every third day) was initiated. On the 6th day of progesterone administration, the embryo transfer was performed, and hormone supplementation was continued until 9–10 weeks’ gestation. Natural cycle (NC) and modified natural cycles (MNC) were monitored for follicular growth and appropriate hormone levels until an endometrial thickness of >7 mm and a dominant follicle >16–17 mm were observed. In NC protocols, ovulation was predicted by changes in serum luteinizing hormone (LH) and confirmed by elevations in progesterone levels. In MNC protocols, a human chorionic gonadotropin (hCG) trigger was administered, and vaginal progesterone (200 mg twice daily) was initiated 1–2 days later. Embryo transfer was performed 1 week after detection of the LH surge (NC) or on the 6th day of progesterone administration (MNC). Progesterone supplementation was continued until 9–10 weeks’ gestation.

### Statistical analysis

All relevant study measures from both the OC dataset and its unique patient identifier-linked OW dataset were summarized using range, mean with SD, median with interquartile range (IQR), or frequency with percentage, as appropriate. Some results were also visualized using boxplots. For all analyses, data from individuals aged 18 to <30 years were pooled; because of small sample sizes at the upper limits of age, and as per the Society of Assisted Reproductive Technology (SART), ages >43 years were pooled for OW outcomes. To test for differences in the age at cryopreservation by year, a Kruskal-Wallis test with Bonferroni-adjusted post hoc pairwise Wilcoxon Rank Sum testing was performed. Both overall and OC age group-specific scatter plots with unadjusted fitted regression lines were also created to examine the relationship between the number of metaphase II (MII) oocytes warmed vs. the number of resulting euploid embryos after PGT-A testing. All final analyses were performed using R version 4.4.1 (R Foundation for Statistical Computing, Vienna, Austria) (21), and a statistically significant *P* value was considered to be < .05, unless adjustment for multiple comparisons was required.

## RESULTS

### Trends in oocyte cryopreservation

Over an 8-year period (2016–2023), a total of 5,900 presumed fertile individuals aged 18 to 45 underwent AMH testing (22). Of those, 3,094 (52.4%) pursued an OC cycle within one year of AMH testing, whereas 2,806 (47.6%) did not proceed with planned OC. Over an 8-year period (2016–2023), 4,659 planned OC cycles were completed for 3,138 patients. Most providers in our practice require AMH testing within 6–12 months of initiating an OC cycle. However, 116 planned OC cycles were performed without recent AMH testing. The mean age at OC cycle start decreased significantly from 36.9 ± 2.8 years in 2016 to 35.0 ± 3.5 years in 2023 (*P*< .001) (Supplemental Fig. 1, available online).

### Characteristics of OW cycles

The first OW cycle was performed in July 2017. Over the subsequent 7 years, 7.9% of patients (n = 249) returned to warm their oocytes. Among patients who had completed at least one OC cycle before 2020 (n = 2,163), allowing for a minimum follow-up period of 4 years, 10.4% (n = 226) returned for OW. The mean age at first OW was 39.9 ± 3.5 years, with a median (IQR) of 40 [39, 42] years. The average interval between OC and OW was 3.4 ± 1.9 years, decreasing with increasing age at OC: 3.9 ± 1.6 years for patients aged 35–37 years, 3.1 ± 2.1 years for those aged 38–40, 2.4 ± 1.6 years for those aged 41–42, and 1.0 ± 1.7 years for patients older than 42 years. Donor sperm was used in 25.1% of cycles and partner sperm in 74.9%.

In total, 4,071 oocytes were warmed across 271 OW cycles (Supplemental Fig. 2). At least one embryo transfer was performed in 144 of 271 OW cycles. Of these, 127 cycles resulted in frozen embryo transfer (FET) of a euploid embryo, 14 cycles in transfer of untested embryos, and 3 cycles in FET after preimplantation genetic testing for monogenic disorders or preimplantation genetic testing for structural rearrangements (PGT-SR). The remaining 127 OW cycles did not advance to embryo transfer because of a variety of clinical, logistical, and patient-related factors. These included no oocyte survival (n = 1), no blastocyst formation (n = 22), exclusively aneuploid blastocysts after PGT-A (n = 27), and transfer to another facility (n = 16). Embryos from 60 OW cycles remain in storage, with patients not yet returning for FET. And, in one case, the patient requested that the embryo be discarded.

### Outcomes of OW cycles

We determined how the age at OC affected OW outcomes. We found that for 168 (62%) warming cycles, oocytes from only one OC cycle were used, and for 103 (38%) warming cycles, oocytes from two or more OC cycles were used. When multiple OC cycles contributed to a single warming cycle, the oldest age at the time of OC was used for further analyses. The average number of MII oocytes warmed per patient per cycle was 15 ± 8.2, with a median (IQR) of 14 (9, 19) (Table 1). Patients younger than 35 years at the time of OC warmed a median (IQR) of 16 (10, 22) MII oocytes per warming cycle, whereas those ≥43 years warmed a median (IQR) of 5 (3, 5) MII oocytes. The overall survival rate of all warmed oocytes was 90.7% ± 13.7%, with the highest survival rate (93.6% ± 11.8%) in patients aged 35–37 years and the lowest rate (79.8% ± 21.4%) in patients aged 43 years and older. Similarly, fertilization rates were highest (78.6% ± 15.0%) in patients aged 35–37 years and lowest (69.0% ± 26.8%) in patients aged 43 years and older. Blastulation rates also peaked (55.4% ± 23.4%) in the 35–37 years age group and were lowest (22.2% ± 43.7%) for those ≥43 years. The mean and median (IQR) age at first embryo transfer were 40 ± 3.6 and 40 (39, 42), respectively. Table 2 and Supplemental Table 1 (available online) demonstrate the association of age at OC and the number of MII oocytes warmed with cumulative ongoing pregnancy/live birth rate (OP/LB) rates across all age groups. For patients ≤40 years of age at the time of OC, the cumulative OP/LB rate was 70.3%, ranging from 58.3% among patients who warmed 1–9 MII oocytes to 81.8% among those who warmed ≥20 MII oocytes. These findings underscore the combined impact of age at OC and the total number of MII oocytes cryopreserved on the likelihood of achieving live birth after OC.

### Age-specific relationship between MII oocytes and embryo ploidy

**FIGURE 1:**
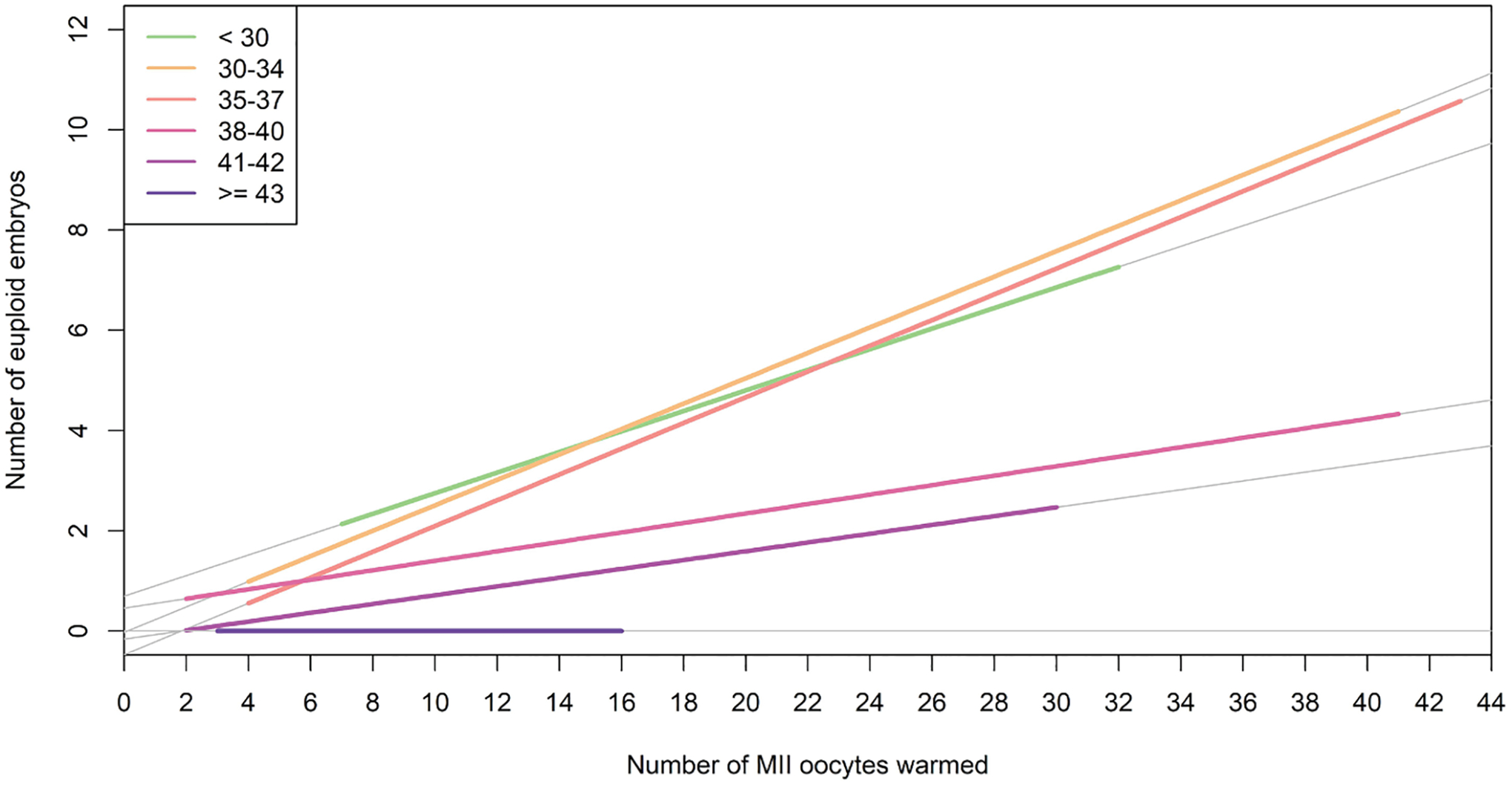
Summary of age-specific euploid embryo outcomes per number MII oocytes warmed. The unadjusted trajectory lines are bolded and colored to reflect the range spanned by the raw data. MII: metaphase II. Klein. 8 years of planned egg freezing. Fertil Steril 2026.

Preimplantation genetic testing for aneuploidy was performed in 88.2% (n = 127/144) of the warming cycles. Given this high rate and the reported correlation between euploid embryo number and live birth prediction (23, 24), we used these data to examine the relationship between the number of warmed MII oocytes and the resulting yield of euploid embryos. Table 3 and Supplemental Table 2 demonstrate the association of age at OC and the number of MII oocytes warmed with euploid embryo yield across all age groups. For patients at ≤40 years of age at the time of OC, the median (IQR) number of euploid embryos increased with cohort size, from 1 [1, 2] in cycles with 1–9 MII oocytes warmed to 5 [2, 8] in those with >20 MII oocytes warmed. We then plotted the number of euploid embryos derived from warmed MII oocytes, stratified by the maximum age at cryopreservation (Fig. 1 and Supplemental Fig. 3). The age groups were selected on the basis of those used by the SART, with added granularity for patients under 35 years. On the basis of the unadjusted regression lines shown (which are emphasized in the range where actual data exist), the relationship between the number of MII oocytes warmed and euploid embryo yield appears to be trimodal. Those in the three lowest age categories (<30, 30–34, and 35–37) have similar trajectories where the slopes of the three lines range from 0.21–0.26, suggesting that, on average, approximately 20%–30% of warmed MII oocytes will become euploid embryos for this age group. On the other hand, those who are in the next two higher age groups (38–40, 41–42) also have similar trajectories compared with each other, but the slopes of the two lines are much flatter and suggest that only approximately 8%–9% of warmed MII oocytes will become euploid embryos. The final age group (≥43) has a trajectory that is perfectly flat, suggesting that, on average, none of their warmed MII oocytes will become euploid embryos; however, this is almost certainly a function of the limited sample size available for this age group.

## DISCUSSION

As demand for planned OC continues to rise, the need for robust patient outcome data becomes increasingly important. In this 8-year single-center cohort, cumulative OP/LB rates after OW were strongly associated with the number of MII oocytes warmed, increasing from 55.6% in cycles with fewer than 10 oocytes to 80% in cycles with >20 oocytes. Oocyte survival, fertilization, and blastulation rates were high, and euploid embryo yield was greater with larger MII oocyte cohorts and younger age at OC. In addition, demographic trends in our cohort reflected a shift toward younger patients undergoing OC and a high utilization of PGT-A. Together, these findings provide important insights into the factors that shape reproductive outcomes after planned OC.

The sociodemographic trends we noted, such as decreasing age at initial OC and the average return age for OW, were similar to those published by prominent academic institutions and international registries (12, 17, 18, 25). We observed a statistically significant decrease in the mean age at cycle start, from 36.9 years at the onset to 35.0 years in the final year of the study, a trend we attribute to a dedicated focus on increasing patient awareness and education about fertility preservation, as well as a focus on affordable pricing relative to most of the established ART programs in our geographic region. Our return/utilization rate for patients who completed OC before 2020, capturing those who froze oocytes at least 4 years before the analysis, was 10.4%, comparable with 10.8% observed in a recent large Austrian study (26). This also aligns with earlier reports indicating return/utilization rates below 15% (14, 18, 27–30). Although it would be difficult to pinpoint an optimal “return rate” for OC, utilizing OC as a strategy for proactive fertility planning may inherently suppress the return rate.

Our study provides valuable insights into OW survival and reproductive outcomes. Our center achieved an overall OW survival rate of 90.7%, surpassing the 78.5% to 81.4% range previously reported in comprehensive meta-analyses and 83.3% in a large international single-center study (14, 18, 26). Our excellent OW survival rate is likely multifactorial, with our specialized focus on OC and high-volume experience likely contributing to improved outcomes. Additionally, our dataset began in 2016, 3 years after the National Institute for Health and Care Excellence established vitrification as the standard of care for fertility preservation (31). In contrast, prior reports included data from before 2013. Our study population utilized donor sperm in 25.1% of OW, a similar utilization rate to the 26.4% published internationally (14). In our experience, most patients undergoing OW elected to use PGT-A. All patients were counseled regarding the risks and benefits of PGT-A (32, 33). Given that 62.6% of patients pursued OC at ≥35 years of age, the high rate of PGT-A utilization likely reflects the risk-averse and proactive reproductive mindset of individuals pursuing OC. The majority selected PGT-A to minimize the likelihood of failed embryo transfers or pregnancy loss and thereby reduce time to viable pregnancy. The utilization of PGT was not consistently stratified in prior studies, making direct comparisons challenging. Our OP/LB rate per OW cycle is similar to that reported in the most extensive single-institution study and recent review of OW outcomes (25, 34).

Our data have significant implications for patient counseling and shared decision-making. The relationship between age at OC and pregnancy outcomes underscores the importance of educating patients on the optimal timing of planned OC. Counseling can be refined by utilizing our MII oocyte to euploid embryo yield stratified by patient age at OC. Although the sample size is limited, this model can help educate patients on the variability inherent in outcomes and support realistic expectations for women considering OC. When planned OC is performed before age 35, a greater number of euploid embryos are available for use, and the success rates of embryo transfer are significantly higher. This notion is supported by the growing literature on planned OC and our findings (35–37). It is also essential to discuss the low utilization rate with patients, as there are significant physical, psychological, and financial implications associated with undergoing planned OC. These factors may directly affect a patient’s medical decision-making.

This study represents one of the largest cohorts of individuals undergoing planned OC and subsequent OW reported by a single center. We provide a more contemporary description of the potential and limitations of OC compared with older publications, including those from the early vitrification era or those reporting outcomes from the slow-freezing method. Our experience contributes robust data to the expanding field of planned OC and fertility preservation. Nonetheless, the study's limitations warrant consideration. First, our return/utilization rate reflects a relatively short follow-up period of 4 years, which limits our ability to capture long-term utilization and pregnancy outcomes. This also introduces potential selection bias, as only patients who returned for OW during this timeframe were included. A more informative interval would arguably extend to at least 10 years. Second, although data from a single center ensures homogeneity in clinical protocols and reporting, the design may limit the generalizability of our findings to other practices. In addition, the sample size was limited at older ages; specifically, the 41–42 and >42 age groups included too few patients to support statistical analysis, making it impossible to draw meaningful conclusions.

## CONCLUSION

As a center dedicated to increasing awareness, education, and access to OC, we are pleased to report our OW outcomes, which significantly complement and expand the current body of published data. In line with ASRM Ethics Committee recommendations, we underscore the importance of reporting clinic-specific outcomes for both OC and OW cycles, as these data are essential for transparent and accurate patient counseling.

### Future directions

Although OC has been a common practice for over a decade, OW remains far less prevalent. It is essential to direct future research toward addressing OC utilization and long-term follow-up. At present, financial limitations are a primary barrier to accessing OC; we are beginning to see an increase in employer-based coverage for fertility preservation services, and how this will impact utilization remains to be seen. Additionally, we should identify the psychosocial factors influencing patients to undergo OC and their decision-making regarding use of their stored oocytes. Lastly, there are well-documented barriers to fertility services for specific populations (38), and it is crucial to address these disparities to ensure equity in OC services. Oocyte cryopreservation is expected to continue growing in popularity and social acceptance, resulting in increased utilization over the coming years. Addressing these topics will enable providers to counsel patients better and maximize the success of fertility preservation.

## Supplementary Material

1

2

Supplemental data for this article can be found online at https://doi.org/10.1016/j.fertnstert.2025.12.003.

## Figures and Tables

**TABLE 1:** Oocyte warming outcomes by age at oocyte cryopreservation.

All oocyte warming cycles						
	All ages (n = 271 cycles)	<35 y (n = 64 cycles)	35–37 y (n = 111 cycles)	38–40 y (n = 74 cycles)	41–42 y (n = 17 cycles)	> = 43 y (n = 5 cycles)
Mean (SD) MII oocytes warmed per patient per cycle	15.0 (8.2)	17.2 (8.6)	15.0 (7.8)	14.6 (8.2)	11.5 (6.7)	6.2 (5.6)
Median (IQR) MII oocytes warmed per patient per cycle	14 (9, 19)	16 (10, 22)	13 (10, 19)	14 (8, 19)	9 (8, 15)	5 (3, 5)
MII oocyte survival rate	90.7% ± 13.7%	92.1% ± 10.2%	93.6% ± 11.8%	86.5% ± 16.6%	88.0% ± 14.9%	79.8% ± 21.4%
Fertilization rate	77.2% ± 16.6%	76.9% ±14.6%	78.6% ± 15.0%	77.8% ± 17.2%	70.2% ± 25.2%	69.0% ± 26.8%
Blastulation rate	49.9% ± 26%	50.1% ± 25%	55.4% ± 23.4%	43.5% ± 25.7%	49.4% ± 32.9%	22.2% ± 43.7%

*Note:* IQR = interquartile range; MII = metaphase II; SD = standard deviation.

Klein. 8 years of planned egg freezing. Fertil Steril 2026.

**TABLE 2:** Cumulative ongoing pregnancy/live birth rate per cycle by age at OC and total number of MII oocytes warmed.

	Any MII warmed	1–9 MII warmed	10–14 MII warmed	15–19 MII warmed	20+ MII warmed
All ages ≤40					
% (95% CI)	70.3 (61.8, 77.6)	58.3 (36.9, 77.2)	66.7 (50.4, 80.0)	67.9 (47.6, 83.4)	81.8 (66.8, 91.3)
n	97/138	14/24	28/42	19/28	36/44
Age <35					
% (95% CI)	80.6 (63.4, 91.2)	75.0 (21.9, 98.7)	60.0 (17.0, 92.7)	77.8 (40.2, 96.1)	88.9 (63.9, 98.1)
n	29/36	3/4	3/5	7/9	16/18
Age 35–37					
% (95% CI)	74.6 (61.8, 84.4)	66.7 (35.4, 88.7)	69.2 (48.1, 84.9)	90.0 (54.1, 99.5)	80.0 (51.4, 94.7)
n	47/63	8/12	18/26	9/10	12/15
Age 38–40					
% (95% CI)	53.9 (37.4, 69.6)	37.5 (10.2, 74.1)	63.6 (31.6, 87.6)	33.3 (9.0, 69.1)	72.7 (39.3, 92.7)
n	21/39	3/8	7/11	3/9	8/11

*Note:* CI = confidence interval; MII = metaphase II; n = number of warming cycles with at least one OP/LB divided by the total number of warming cycles; OC = oocyte cryopreservation; OP/LB = ongoing pregnancy/live birth rate calculated on the basis of a patient having at least one ongoing pregnancy or live birth.

Klein. 8 years of planned egg freezing. Fertil Steril 2026.

**TABLE 3:** Number of euploid embryos per cycle by age at OC and total number of MII oocytes warmed.

	1–9 MII warmed	10–14 MII warmed	15–19 MII warmed	20+ MII warmed
All ages ≤40				
Median (IQR)	1 (1, 2)	2 (1, 3)	2 (1, 4)	5 (2, 8)
n	45	59	51	59
Age <35				
Median (IQR)	2 (1, 2)	2 (2, 3)	4 (2, 6)	7 (3, 8)
n	8	11	14	21
Age 35–37				
Median (IQR)	1 (1, 2)	3 (2, 4)	2 (1, 4)	6 (4, 9)
n	24	31	23	22
Age 38–40				
Median (IQR)	0 (0, 1)	1 (1, 3)	2 (1, 3)	2 (1, 4)
n	13	17	14	16

*Note:* IQR = interquartile range; MII = metaphase II; n = number of warming cycles that underwent PGT-A; OC = oocyte cryopreservation; PGT-A = preimplantation genetic testing for aneuploidy.

Klein. 8 years of planned egg freezing. Fertil Steril 2026.
