# Supplementary material for: Freezing first: insights from 8 years of planned oocyte cryopreservation at an “egg freezing clinic”
Source: Fertil Steril. Author manuscript; Available in PMC 2026 Jul 1. (PMC13322467; doi:10.1016/j.fertnstert.2025.12.003)
Supplement: 1 [file NIHMS2187415-supplement-1.pdf]

Supplementary Figure 1 . Age at oocyte cryopreservation cycle start for each year, 2016 – 2023

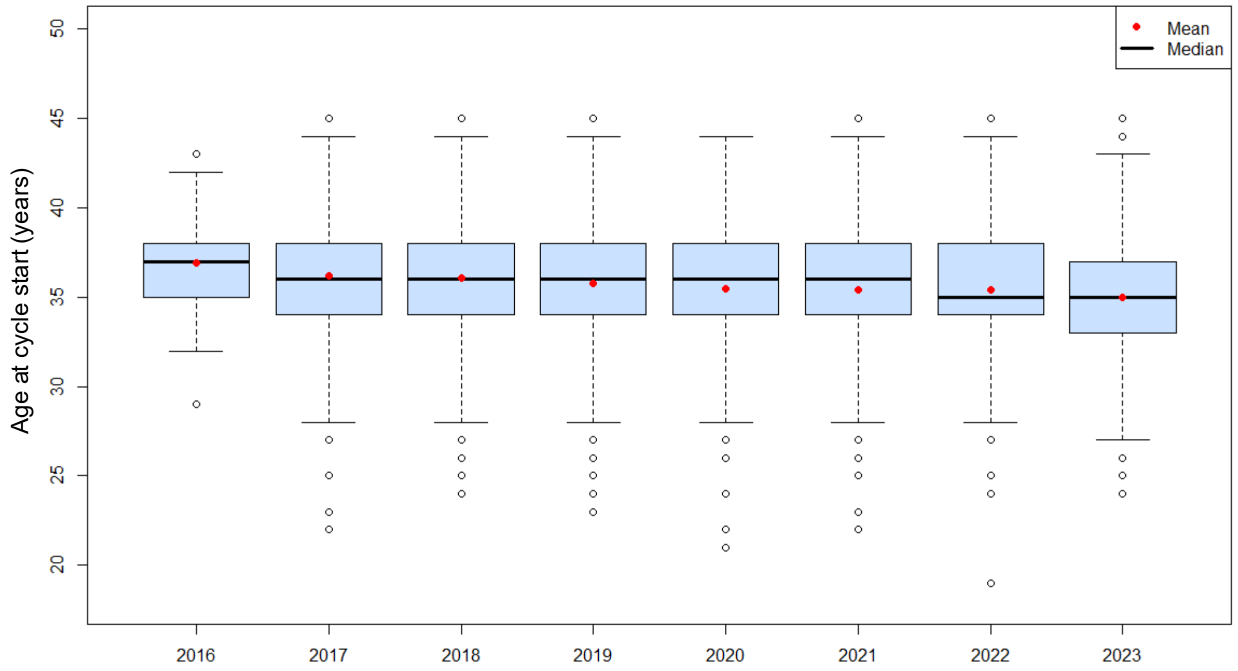

Supplementary Figure 2. Overview of oocyte warming cycles

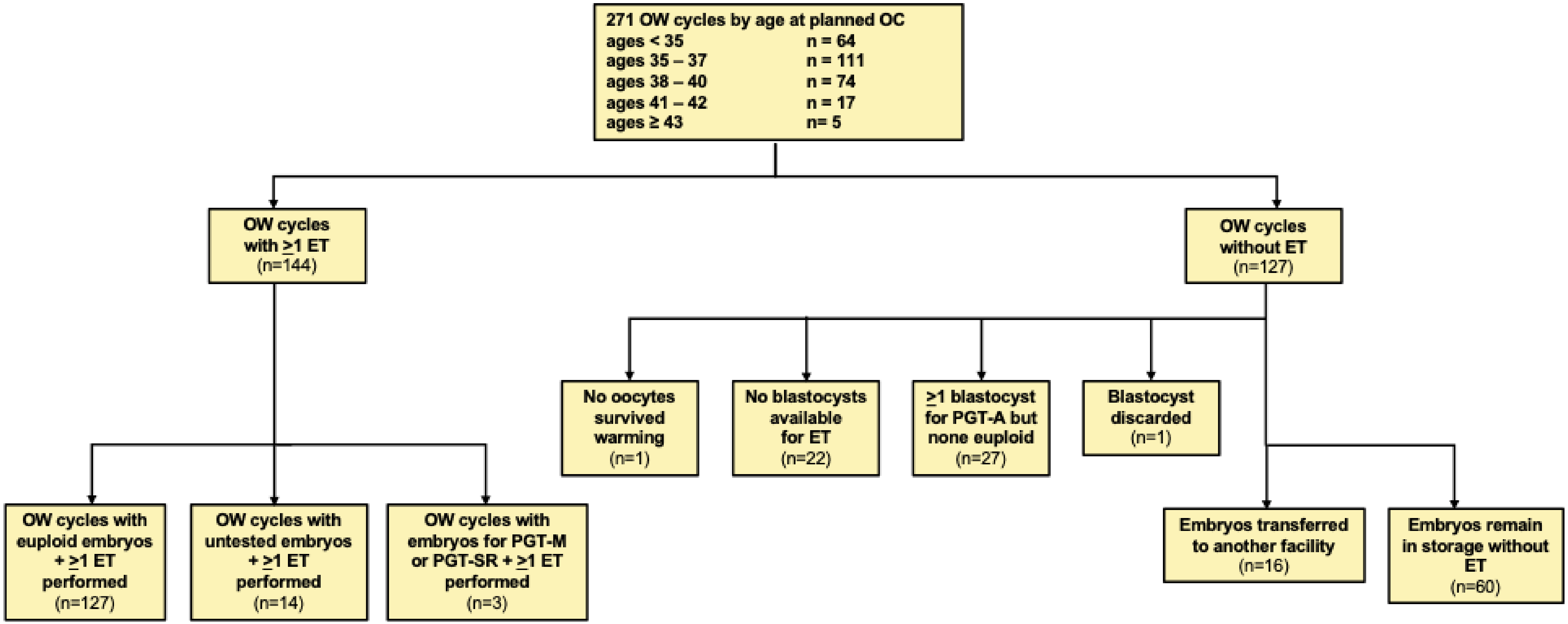

Supplementary Figure 2: Overview of oocyte warming cycle outcomes.
ET: embryo transfer; FET: frozen embryo transfer; OW: oocyte warming; PGT-A: preimplantation genetic testing-aneuploidy; PGT-M: preimplantation genetic testing-monogenic disorders; PGT-SR: preimplantation genetic testing-structural rearrangements

Supplementary Figure 3. Age-specific euploid embryo outcomes per number MII oocytes warmed

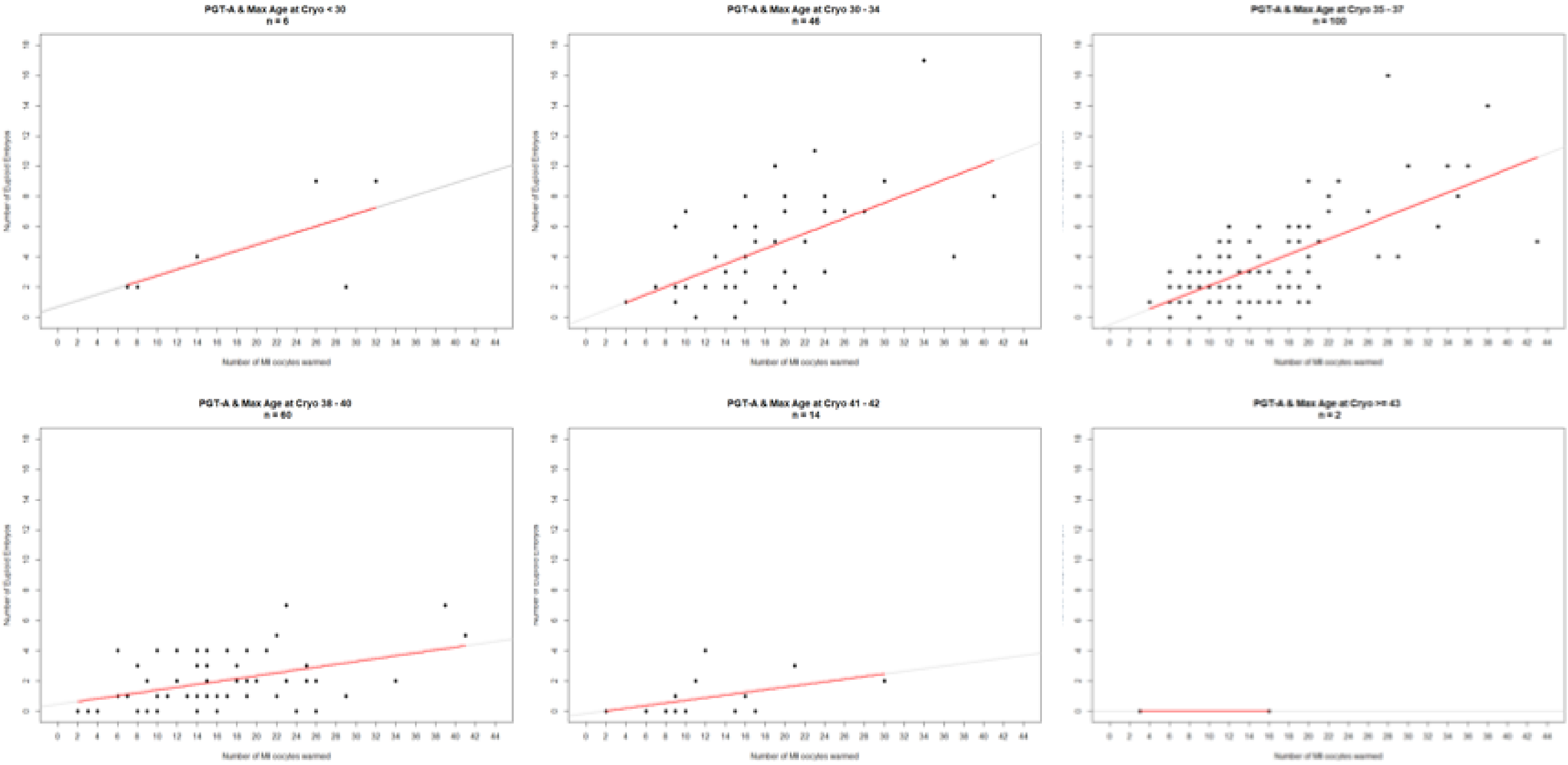

Supplementary Figure 3. Age-specific euploid embryo outcomes per number MII oocytes warmed.
PGT-A: preimplantation genetic testing-aneuploidy; MII: metaphase II
