# Supplementary material for: Freezing first: insights from 8 years of planned oocyte cryopreservation at an “egg freezing clinic”
Source: Fertil Steril. Author manuscript; Available in PMC 2026 Jul 1. (PMC13322467; doi:10.1016/j.fertnstert.2025.12.003)
Supplement: 2 [file NIHMS2187415-supplement-2.docx]

**Supplementary Table 1: Cumulative ongoing pregnancy/live birth rate per cycle by age at OC and total number of MII oocytes warmed for patients > 40 years**

|  | **Any MII warmed** | **1 – 9 MII warmed** | **10 – 14 MII warmed** | **15 – 19 MII warmed** | **20+ MII warmed** |
| --- | --- | --- | --- | --- | --- |
| **All Ages > 40** |  |  |  |  |  |
| % (95% CI) | 33.3 (6.0, 75.9) | 33.3 (1.8, 87.5) | 0.0 (0.0, 94.5) | 100.0 (5.5, 100.0) | 0.0 (0.0, 94.5) |
| n | 2/6 | 1/3 | 0/1 | 1/1 | 0/1 |
|  |  |  |  |  |  |
| **Age 41-42** |  |  |  |  |  |
| % (95% CI) | 40.0 (7.3, 83.0) | 50.0 (9.5, 90.5) | 0.0 (0.0, 94.5) | 100.0 (5.5, 100.0) | 0.0 (0.0, 94.5) |
| n | 2/5 | 1/2 | 0/1 | 1/1 | 0/1 |
|  |  |  |  |  |  |
| **Age >=43** |  |  |  |  |  |
| % (95% CI) | 0.0 (0.0, 94.5) | 0.0 (0.0, 94.5) | NA | NA | NA |
| n | 0/1 | 0/1 | NA | NA | NA |

OP/LB: ongoing pregnancy/live birth rate calculated based on a patient having at least one ongoing pregnancy or live birth; CI: confidence interval; OC: oocyte cryopreservation; MII: metaphase II.

n: number of warming cycles with at least one OP/LB divided by the total number of warming cycles

**Supplementary Table 2: Number of euploid embryos per cycle by age at OC and total number of MII oocytes warmed for patients > 40 years**

|  | **1 – 9 MII warmed** | **10 – 14 MII warmed** | **15 – 19 MII warmed** | **20+ MII warmed** |
| --- | --- | --- | --- | --- |
|  |  |  |  |  |
| **All Ages > 40** |  |  |  |  |
| Median [IQR] | 0 [0, 0] | 2 [1, 3] | 0 [0, 0] | 2 [2, 2] |
| n | 7 | 3 | 4 | 2 |
|  |  |  |  |  |
| **Age 41-42** |  |  |  |  |
| Median [IQR] | 0 [0, 0] | 2 [1, 3] | 0 [0, 0] | 2 [2, 2] |
| n | 6 | 3 | 3 | 2 |
|  |  |  |  |  |
| **Age >=43** |  |  |  |  |
| Median [IQR] | 0 [0, 0] | NA | 0 [0, 0] | NA |
| n | 1 | 0 | 1 | 0 |

OC: oocyte cryopreservation; MII: metaphase II

n: number of warming cycles that underwent PGT-A
